# High Blood Pressure prevention and control: from evidence to action

**DOI:** 10.25100/cm.v49i2.3940

**Published:** 2018-06-30

**Authors:** Lena Barrera

**Affiliations:** Control and Prevention of Chronic Diseases research group. Departamento de Medicina Interna, Facultad de Salud, Universidad del Valle. Cali, Colombia

The Pan American Health Organization (PAHO) with the World Hypertension League has established the 17^th^ May to promote the prevention and control of High Blood Pressure (HBP) ^1^. Currently nearly 1.13 billion of adults suffer from HBP (blood pressure >=140/90 mm Hg) worldwide ^2^. While HBP prevalence decreased in high income countries (HIC) between 1975 and 2015, the opposite trend was observed in low- and middle- income countries (LMIC) ^3^. Particularly, in Latin American and Caribbean countries the prevalence decreased from 40.6% to 26.8% and from 26.8% to 19.4% for men and women between 45 to 49 years old respectively ^3^. However, in 2015, HBP accounted for 8.9 of the total of disability adjusted life years (DALYS) and was associated with 4.9 million, 2.0 million and 1.5 million deaths due to ischemic heart disease, hemorrhagic stroke and ischemic stroke respectively ^4^. Therefore, HBP is the leading cardiovascular risk factor worldwide. In Colombia, the last National Health Survey reported a global prevalence of 22.8% and nearly 60% for those between 60 and 69 years in 2007 ^5^. 

High Blood Pressure have been clearly associated with biological risk factors and environment conditions. Studies carried out in western societies have revealed that whereas systolic blood pressure rises continually with age starting at 30 years; diastolic blood pressure rises continually until 50 years and then remains stable or slightly decreases ^6^. HBP has been found positively associated with parental high blood pressure, pre-eclampsia, low birth and increases in body mass index ^7^. While increased physical activity and low salt consumption have been found negatively correlated with HBP ^7^. At macro level, urbanization and pollution has been linked to higher prevalence of HBP ^8^ ^,^ ^9^. Lower socio-economic status and lower education have been reported associated with higher prevalence of HBP, mainly in HICs although little evidence has been documented in LMICs ^10^ ^,^ ^11^. Generally, people living in rural areas working in agriculture and manual activities have lower blood pressure ^12^.

High Blood Pressure and related cardiovascular diseases are preventable and interventions at both population and individual level have effectively reduced the burden of HBP ^7^. Government regulations to lower salt content added to processed food is one the most supported population interventions ^13^. A decline of 30% in sodium intake has been associated with a reduction of 10 mm Hg in the average population of systolic and diastolic blood pressure ^14^. At individual level the use of pharmacological and non-pharmacological interventions has clearly demonstrated the benefits of lowering BP. Randomized controlled trials have shown that a reduction of 10 mm Hg BP in systolic blood pressure results in 17% and 27% lower probability of suffering from coronary heart disease and stroke respectively ^15^. Increased physical activity, low salt intake, loss weight, moderate alcohol consumption and the DASH or Meditarrean diets decrease systolic blood pressure by 6.0 mm Hg on average ^16^.

In spite of having ample evidence on the effectiveness of prevention and treatment for HBP, only 50% of hypertensive people have BP controlled (BP<140/90 mm Hg) worldwide ^17^. Whereas in 2010, the HIC rates of awareness, treatment and control were 67.0% 55.6%, 50.4% respectively, the equivalent in LMIC rates were 37.9%, 29.0% and 26.3% ^17^. Similar results have been provided by the PURE study which included population from Latin American countries. In Colombia, by example, the PURE study showed 51.9% awareness of HBP, 77.5% were on antihypertensive treatment and 37.1% had HBP controlled ^18^. 

Tackling the burden of HBP is a complex public health issue. A comprehensive and life course approach is needed to impact the multifactorial factors that accounting for the prevalence, access to treatment and the control of HBP ^7^ ^,^ ^19^. The approach should include an adequate pregnancy care, the promotion of healthy behaviors for all population such as low salt intake, physical activity, weight loss and moderate alcohol consumption, the early detection of increases in BP, universal access to pharmacological and non-pharmacological interventions and the monitoring of HBP. To accomplish the objectives the health sector needs to work with other social sectors such as the educational, food makers and social media as well. 

We celebrate the “Know your numbers” 2018 PAHO initiative to improve the control and prevention of HBP ^1^. Each adult should be prompted to know his BP level. Standardized accurate measurement of BP should be available for all population. Health workers need to be trained on the method of measurement BP. Health services also could monitor the numbers of adults that have been checked BP among those for whom health care has been provided. Having BP measured, people should be encouraged to adapt healthy behavior to prevent increases in blood pressure levels and HPB related diseases. Certainly, the measurement of BP will lead to increases in the rate of HBP awareness and so knowing the roots of the HBP burden. 
