# Supplementary material for: High Blood Pressure prevention and control: from evidence to action
Source: Colomb Med (Cali). 2018 Jun 30;49(2):137–8. doi: 10.25100/cm.v49i2.3940 (PMC6084923; doi:10.25100/cm.v49i2.3940)
Supplement: Related texts

# Editorial: Presión arterial elevada: de la evidencia a la acción

La Organización Panamericana de la Salud (OPS) y la Liga Mundial de Hipertensión han establecido el 17 de mayo como el día internacional para promover la prevención y el control de la Presión Arterial Elevada (PAE) ^1^. Actualmente cerca de 1.13 billones de adultos sufren de PAE (presión arterial >=140/90 mm Hg) alrededor del mundo ^2^. Mientras la prevalencia de PAE disminuyó en países de alto ingreso entre 1975 y 2015, una tendencia opuesta se observó en los países de mediano y bajo ingreso en el mismo período ^3^. Particularmente, en los países de América Latina y del Caribe la prevalencia disminuyó de 40.6% a 26.8% y de 26.8% a 19.4% en hombres y mujeres entre edades de 45 a 49 años respectivamente ^3^. Sin embargo, en el 2015, la PAE representó el 8.9% de los total de años de vida perdidos potencialmente perdidos por discapacidad y mortalidad prematura (AVPP) y fue asociado con 4.9 millones, 2.0 millones y 1.5 millones de muertes debidas a enfermedad isquémica cardíaca, enfermedad cerebro-vascular hemorrágica y enfermedad cerebro-vascular isquémica respectivamente ^4^. Por lo tanto, la PAE es el principal factor de riesgo cardiovascular en el mundo. En Colombia, la última Encuesta Nacional de Salud reportó una prevalencia global de 22.8% y cerca del 60% para la población con edades entre 60 y 69 años en el 2017 ^5^. 

La PAE ha sido claramente asociada con factores de riesgo biológicos y condiciones del medio ambiente. Estudios llevados en sociedades occidentales han mostrado que mientras la presión arterial aumenta en forma constante con la edad a partir de los 30 años, la presión arterial diastólica aumenta continuamente hasta la edad de 50 años y luego permanece estable o desciende en forma moderada ^6^. La PAE se ha asociado en forma positiva con antecedente familiar de PAE, pre-eclampsia, bajo peso al nacer y aumento en el índice de masa corporal ^7^. En forma opuesta, el incremento en actividad física y el bajo consumo de sal se han correlacionado en forma negativa con la PAE ^7^. A nivel macro, la urbanización y la contaminación ambiental han sido asociados con alta prevalencia de PAE ^8^^,^^9^. El bajo nivel socio-económico y el bajo nivel educativo han sido asociados con una alta prevalencia de PAE principalmente en países de alto ingreso aunque un poco menos de evidencia ha sido documenta en países de mediano y bajo ingreso ^10^^,^^11^. Generalmente, personas residentes en zonas rurales trabajando en agricultura y labores manuales presentan niveles bajos de presión arterial ^12^. 

La PAE y las enfermedades cardiovasculares relacionadas son prevenibles y tanto las intervenciones a nivel individual como poblacional han efectivamente reducido la carga de enfermedad asociada a PAE ^7^. Las regulaciones gubernamentales orientadas a la reducción en el contenido de sal en los productos alimenticios manufacturados es una de las intervenciones con mayor evidencia ^13^. Una caída del 30% en el consumo de sodio ha sido asociada con una reducción de 10 mm Hg en el promedio poblacional de la presión arterial sistólica y diastólica ^14^. A nivel individual el uso de intervenciones farmacológicas y no farmacológicas han claramente demostrado el beneficio de reducir los niveles de presión arterial. Los ensayos clínicos randomizados han mostrado que una reducción de 10 mm Hg en la presión arterial sistólica conduce a una reducción del 17% y del 27% en la probabilidad de sufrir un evento coronario o un evento cerebro-vascular respectivamente ^15^. La actividad física intensa, el bajo consumo de sal, la reducción del peso, el moderado consumo de alcohol, y la dieta DASH o la dieta del Mediterráneo disminuyen la presión arterial en 6 mm Hg en promedio ^16^. 

A pesar de la amplia evidencia disponible acerca de la efectividad de prevenir y tratar la PAE, solo el 50% de la personas con PAE tienen la presión arterial controlada (Presión arterial <140/90 mm Hg) a nivel mundial ^17^. Mientras en el año 2010, la tasas de conocimiento, tratamiento y control de la PAE fueron 67.0%, 55.6% y 50.4% respectivamente en los países de alto ingreso, en los países de bajo y mediano ingreso estas tasas fueron 37.9%, 29.0% y 26.3% respectivamente ^17^. Resultados similares fueron reportados por el estudio PURE en el cual se incluyó población de países latino-americanos. Para Colombia, por ejemplo, el estudio PURE mostró una tasa de conocimiento de PAE del 51.9%, y que el 77.5% de la población con PAE recibía tratamiento antihipertensivo y de esta el 37.1% se encontraba controlada ^18^. 

Reducir la carga de enfermedad atribuida a la PAE es una situación compleja en salud pública. Es necesario desarrollar una estrategia exhaustiva e integral basada en un enfoque de curso de vida que permita impactar los múltiples factores que determinan la prevalencia, el acceso al tratamiento y el control de la PAE ^7^^,^^19^. Este enfoque incluye un adecuado cuidado prenatal, la promoción de hábitos de vida saludable para toda la población como el bajo consumo de sal, la realización de actividad física, la pérdida de peso, el moderado consumo de alcohol y la detección temprana del aumento de la PAE, el acceso universal a las intervenciones farmacológicas y no farmacológicas, y el monitoreo de la PAE. El desarrollo de estas estrategias requiere el trabajo integrado entre el sector salud y otros sectores de la sociedad como educación, productores de alimentos y los medios masivos de comunicación.

Nosotros celebramos la campaña “Conoce tus números” que este año 2018 la OPS está promoviendo para mejorar la prevención y el control de la PAE ^1^. Cada adulto debe ser motivado a conocer su nivel de presión arterial. La toma estandarizada y precisa de la presión arterial debe ser accesible a toda la población. Los trabajadores de la salud necesitan ser entrenados en el método para tomar adecuadamente la presión arterial. Adicionalmente, los servicios de salud podrían monitorizar la cantidad de personas a quienes se les ha tomado la PAE entre la población que recibe atención en salud al interior de cada centro. Luego de haber tomado la presión arterial, las personas deben ser motivadas a adaptar comportamientos saludables que prevengan el aumento de la presión arterial y las enfermedades relacionadas con la PAE. Sin lugar a dudar, la toma de la presión arterial conducirá a un incremento en la tasa de conocimiento de la PAE y las raíces de la carga de enfermedad relacionada con la PAE.
